# Na^+^/Ca^2+^ Exchanger 1 in Airway Smooth Muscle of Allergic Inflammation Mouse Model

**DOI:** 10.3389/fphar.2018.01471

**Published:** 2018-12-13

**Authors:** Jiexia Wen, Xiangcai Meng, Bin Xuan, Tao Zhou, Heran Gao, Hui Dong, Yimin Wang

**Affiliations:** ^1^Department of Central Laboratory, The First Hospital of Qinhuangdao, Hebei Medical University, Qinhuangdao, China; ^2^Department of General Surgery, The First Hospital of Qinhuangdao, Hebei Medical University, Qinhuangdao, China

**Keywords:** airway smooth muscle, intracellular Ca^2+^, NCX1, allergic inflammation, asthma

## Abstract

Cytosolic free Ca^2+^ ([Ca^2+^]_cyt_) is essential for airway contraction, secretion and remodeling. [Ca^2+^]_cyt_ homeostasis is controlled by several critical molecules, one of which is the Na^+^/Ca^2+^ exchanger 1 (NCX1) in the plasma membrane. Since little is currently known about NCX1 in the airway smooth muscle and its involvement in airway diseases, the present study was designed to investigate the expression and function of NCX1 in normal airway smooth muscle and its relevance to airway inflammation. Western blot analysis, tracheal smooth muscle contraction, and [Ca^2+^]_cyt_ measurements were performed in mouse tracheal smooth muscle tissues and primary airway smooth muscle cell cultures. Additional studies were performed in a mouse model of allergic airway inflammation. Our data showed that NCX1 proteins were expressed in the human bronchial smooth muscle cells (HBSMCs), murine airway and whole lung. Carbachol raised [Ca^2+^]_cyt_ in mouse tracheal smooth muscle cells and induced murine tracheal contraction, all of which were significantly attenuated by KB-R7943, a selective NCX inhibitor. Removal of extracellular Na^+^ increased [Ca^2+^]_cyt_ in HBSMCs and mouse tracheal SMCs, which was dependent on extracellular Ca^2+^ and sensitive to KB-R7943. TNF-α treatment of HBSMCs significantly upregulated mRNA and protein expression of NCX1 and enhanced NCX activity. Finally, KB-R7943 abolished the airway hyperresponsiveness to methacholine in an ovalbumin-induced mouse model of allergic airway inflammation. Together, these findings indicate that NCX1 in airway smooth muscle may play an important role in the development of airway hyperresponsiveness, and downregulation or inhibition of NCX1 may serve as a potential therapeutic approach for asthma.

## Introduction

Asthma is a major public health issue afflicting as many as 22 million people in the United States and approximately 300 million worldwide (Bateman et al., 2008). The consequences, including the loss productivity, emergency room visits, and hospitalizations, as well as direct and indirect costs, are profound (McCracken et al., 2017). Asthma is associated with airway inflammation, airway hyperresponsiveness and bronchospasm. Airway obstruction due to bronchoconstriction and hypersecretion of mucus is a major cause for acute respiratory incapacity in patients with asthma (Contoli et al., 2015; Fehrenbach et al., 2017). Airway smooth muscle can undergo functional change and hypertrophy, which contributes to the development of persistent airway obstruction and increased non-specific airway hyperresponsiveness in chronic severe asthma (Nadel and Busse, 1998; Cohn et al., 2004; Fehrenbach et al., 2017). Therefore, airway smooth muscle is critical in the development of asthma.

A rise in ([Ca^2+^]_cyt_) in airway smooth muscle cells is essential for airway contraction, secretion and remodeling; therefore abnormalities of [Ca^2+^]_cyt_ homeostasis in airway cells may play a key role in the pathogenesis of asthma. Although multiple molecules and proteins control [Ca^2+^]_cyt_ homeostasis, one of these is the plasma membrane Na^+^/Ca^2+^ exchanger (NCX). NCXs are actually a family of membrane transporters that can exchange Na^+^ and Ca^2+^ (or K^+^) in either direction depending on the transmembrane electrochemical gradient of Na^+^. Two sub-families of these exchange proteins have been described in mammalian cells (Blaustein and Lederer, 1999; Visser and Lytton, 2007; Khananshvili, 2013): one in which Ca^2+^ movement is dependent only on Na^+^ (NCX1-3), and the other in which Ca^2+^ transport is also dependent on K^+^ (NCKX1-6). NCXs have been described in mammalian tissues for over three decades, but very little is currently known about the expression and function of these proteins in airway smooth muscle (or other cell types in the airway). Although NCX-mediated Ca^2+^ fluxes may contribute to enhanced [Ca^2+^]_cyt_ regulation in airway inflammation (Sathish et al., 2011), the possible involvement of Na^+^/Ca^2+^ exchange in airway diseases, such as asthma, is unexplored.

The current study was designed to examine the expression and function of Na^+^/Ca^2+^ exchange protein 1 (NCX1), a major isoform of NCXs in human airway smooth muscle cells (Liu et al., 2010), and to explore the role of NCX1 in airway diseases such as asthma. We performed biochemical and functional studies of human and murine airway smooth muscle cells, tested murine tracheal tissues, and developed an allergic airways disease model in mice to examine: (1) the expression of NCX1 mRNA and proteins in human and murine airway smooth muscle cells; (2) the role of NCX1 in regulating [Ca^2+^]_cyt_ homeostasis in these cells; (3) the effect of the proinflammatory cytokine, TNF-α, on the expression and function of NCX1 proteins in primary cultures of HBSMCs in a cell model of allergic airway inflammation; (4) the role of NCX1 proteins on airway hyperresponsiveness in a mouse model of allergic airway inflammation. Some of these data have been published previously in an abstract form (Dong et al., 2009).

## Materials and Methods

### Preparation of Trachea and Measurement of Tracheal Contraction

All animal experiments were carried out in accordance with the First Hospital of Qinhuangdao Guide for the Care and Use of Laboratory Animals. Adult C57BL/6 mice of both sexes were housed in an animal care room with a 12-h light-dark cycle and were allowed free access to food and water. Mice were killed by cervical dislocation after anesthesia with halothane at about 8 weeks of age. The trachea was rapidly removed and placed in ice-cold normal physiological salt solution (PSS) of the following composition: NaCl 118 mM; KCl 4.7 mM; CaCl_2_ 2.5 mM; KH_2_PO_4_ 1.2 mM; MgSO_4_ 1.2 mM; NaHCO_3_ 12.5 mM; dextrose 11.1 mM. The pH of the PSS after saturation with 95% O_2_ + 5% CO_2_ gas mixture was 7.4. Adherent connective tissue was removed from trachea using a surgical microscope. Epithelial cells were removed from all tracheas by repeatedly passing a stainless steel cannula of appropriate size through the lumen.

Isometric force development was recorded in segments of tracheal rings (5 mm) by multichannel recorder, as previously described (Dong et al., 1997, 2000). Briefly, two tungsten wires (0.5 mm diameter) were inserted through the lumen of the trachea. One wire was then attached to a force transducer and the other connected to a micrometer. The trachea was placed in a 10 ml organ bath containing gassed PSS. Tracheal tissues were routinely allowed to equilibrate for 1 h before the start of the experiments. Isometric tension of trachea was recorded with a force displacement transducer (Grass FT03) coupled to a Grass polygraph (model 7E).

### Airway Smooth Muscle Cell Preparation and Primary Culture

Primary cultures of murine tracheal smooth muscle cells were prepared from adult C57BL/6 mice. The isolated trachea was placed in cold PSS bubbled with 95% O_2_ + 5% CO_2_. After adherent connective tissue was removed from the trachea, they were cut longitudinally and epithelial cells were also removed by mildly rubbing the luminal surface with a cotton swab. The tracheal tissues were washed three times with fresh PSS and then cut with fine scissors into small pieces (3 mm × 4 mm). These pieces were seeded onto P-100 plastic dishes (MatTek Corporation, Ashland, MA, United States), and cultured in Dulbecco’s modified Eagle’s medium (DMEM) containing 10% fetal bovine serum, 100 U/ml penicillin G and 100 mg/ml streptomycin at 37°C in a humidified incubator equilibrated with 5% CO_2_ and 95% air. After 4–5 days in culture, the small pieces of tracheal tissue were removed and single cells were trypsinized and re-plated on 10-mm diameter circular, glass cover slips that were pre-coated with 1 mg/ml poly-D-lysine (Sigma).

The collection and use of all human samples were approved by the Institute’s Human Ethics Committee of the First Hospital of Qinhuangdao and in accordance with the Declaration of Helsinki. Patients undergoing lobectomy or lung transplantation for lung cancer who had no evidence of asthma were the source of lung tissues for preparing primary cultures of HBSMCs. Lung tissues were removed from patients in the operating room, immediately placed in cold (4°C) saline, and taken to the laboratory for dissection. Bronchi were isolated from the lung tissues, and the smooth muscle segment was digested with 2.0 mg/ml collagenase and 0.5 mg/ml elastase at 37°C. The HBSMCs were cultured in smooth muscle growth medium (Lonza; Walkersville, MD, United States), which consisted of smooth muscle basal medium, 5% FBS, 5 μg/ml insulin, 2 ng/ml human fibroblast growth factor, and 0.5 ng/ml human epidermal growth factor, for 5–7 days before each experiment.

Cell number was determined by a hemocytometer. The normalized cell number by size of the Petri dishes or cover slips (cells/cm^2^) was used to compare cell growth rate. Cell viability was determined by using 0.45% trypan blue (Sigma Chemical).

### [Ca^2+^]_cyt_ Measurement With a Digital Imaging System

[Ca^2+^]_cyt_ in airway smooth muscle cells was measured by Fura 2-AM fluorescence ratio digital imaging as described previously (Smith et al., 2006). Briefly, airway smooth muscle cells grown on cover slips for at least 24 h were loaded with 5 μM Fura 2-AM (dissolved in 0.01% Pluronic F-127 plus 0.1% DMSO in PSS) at room temperature (22–24°C) for 50 min, then washed in normal PSS for at least 20 min. Thereafter, the cover slips with airway smooth muscle cells were mounted in a perfusion chamber on a Nikon microscope stage. Cells were initially superfused with PSS for 5 min (at room temperature), and then switched to Ca^2+^-free or Ca^2+^-containing solutions with different drugs. The ratio of Fura 2-AM fluorescence (510-nm light emission excited by 340- and 380-nm illuminations) from the cells, as well as background fluorescence, was collected at room temperature (22°C) with the use ofa 40 × Nikon UV-Fluor objective and an intensified CCD camera (ICCD200). The fluorescence signals emitted from the cells were monitored continuously using a MetaFluor Imaging System (Universal Imaging, Corporation, Downingtown, PA, United States) and were recorded in an IBM-compatible computer for later analysis. [Ca^2+^]_cyt_ was calculated from Fura 2-AM fluorescent emission excited at 340 and 380 nm (F_340_/F_380_) using the ratio method based on the equation: [Ca^2+^]_cyt_ = *K*_d_ × (Sf_2_/Sb_2_) × (R-R_min_)/(R_max_-R), where *K*_d_ (225 nM) is the dissociation constant for Ca^2+^, R is the measured fluorescence ratio, and R_min_ and R_max_ are minimal and maximal ratios, respectively (Grynkiewicz et al., 1985).

### Western Blot Analysis of NCX1 Proteins

Proteins were extracted from mouse heart, lung and tracheal tissues, or from HBSMC by homogenization on ice in 500 μl of lysis buffer containing: 20 mM Tris⋅HCl (pH 7.5), 150 mM NaCl, 1 mM disodium EDTA, 1 mM EGTA, 2.5 mM sodium pyrophosphate, 1 mM β-glycerophosphate, 1 mM sodium orthovanadate, 1% Triton X-100, and complete protease inhibitor cocktail (AEBSF 104 mM, aprotinin 0.08 mM, leupeptin 2 mM, bestatin 4 mM, pepstatin 1.5 mM, and E-64 1.4 mM; Sigma, St. Louis, MO, United States). Equal amounts of protein, as determined by Lowry assay (D_c_ assay; Bio-Rad, Hercules, CA, United States), were combined with 2× Laemmli sample buffer. Samples were not boiled because this hydrophobic, membrane-bound protein was found to aggregate with boiling. Proteins were separated by electrophoresis on 4–15% SDS-PAGE and transblotted to nitrocellulose membranes. The protein-bound nitrocellulose membranes were first incubated 30 min at room temperature in blocking buffer containing 2% non-fat dry milk in distilled water. Nitrocellulose membranes were incubated with R3F1 monoclonal antibody to NCX1 (Swant, Bellinzona, Switzerland) diluted in blocking buffer (1:5,000) overnight at 4°C and then rinsed for 1 h with a wash buffer containing 20 mM Tris, pH 7.5, 500 mM NaCl, and 1% Tween 20. The membranes were then incubated with horseradish peroxidase-conjugated donkey anti-mouse IgG antibody for 30 min at room temperature and washed for 1 h with agitation, changing the wash buffer every 15 min. Protein bands were visualized with ECL Plus detection reagents (Amersham and Pharmacia, Piscataway, NJ, United States), with NCX1 bands occurring at 120 and 70 kDa.

### RNA Extraction and Quantitative Reverse-Transcription PCR

Total cellular RNA was isolated from HBSMCs by the single-step guanidinium thiocyanate method using Trizol (Invitrogen, Carlsbad, CA, United States) according to the manufacturer’s instructions. To remove residual DNA, RNA samples were treated with RNAse-free DNAse and purity was confirmed by 260/280 ratios. RNA was reverse-transcribed using Superscript II (Invitrogen, Carlsbad, CA, United States). Real time PCR was performed in an M × 3000P real-time PCR system (Stratagene, La Jolla, CA, United States) using SYBR Green I dye as the detection format. A 16 μl reaction volume contained 16 ng of cDNA, 100 nM primers, using 1× of iQ SYBR Green super mix (Bio-Rad Laboratories; Hercules, CA, United States). The primers for human NCX1 were (forward) TGTGCATCTCAGCAATGTCA and (reverse) TTCCTCGAGCTCCAGATGTT. Glyceraldehyde 3-phosphate dehydrogenase (GAPDH) was used as a control (housekeeping gene) to control for potential differences in RNA input. The primers for GAPDH were (forward) ACAGTCAGCCGCATCTTCTT and (reverse) TGGAAGATGGTGATGGGATT. Data were analyzed by determining the threshold cycle time (Ct) and normalizing NCX1 Ct to GAPDH Ct using the ΔΔCt method (Radonic et al., 2004).

### Mouse Model of Allergic Airway Inflammation

C57BL/6 mice (The Jackson Laboratory, Bar Harbor, ME, United States) 6–8 weeks old were immunized and challenged over a 21 day protocol modified from a previously reported method (Velasco et al., 2005). On days 0 and 7 mice were immunized with intraperitoneal injections of 50 μg of LPS-free ovalbumin (Profos AG, Regensburg, Germany) absorbed by 1 mg of alum (Sigma-Aldrich, St. Louis, MO, United States) in 100 μl of PBS. Subsequently on days 17, 18, 19, and 20 mice were challenged with ovalbumin intratracheal instillations of 20 μg in 50 μl PBS delivered via direct visualization of the vocal cords and using a gel loading micropipette.

On day 21, anesthesia was induced with 0.5 mg/0.5 mg ketamine/xylazine (Vedco Inc., St. Joseph, MO, United States) in 50 μl PBS and 3% Isoflurane (Baxter; Deerfield, IL, United States). Mice were then intubated using a 20 ga, 1 inch intravenous catheter (Optiva, Johnson & Johnson) guided by a PE10 stylet placed under direct visualization of the vocal cords. Mice were then placed on a mechanical ventilator (Flexivent; Scireq, Montreal, QC, Canada), and ventilated quasi-sinusoidally at 150 breaths per minute, a tidal volume of 6 μl/g and 4 cm H_2_O of PEEP. The proper placement of the endotracheal tube was verified by the generation of negative inspiratory airway pressures and expiratory gas output via the PEEP trap. The animals were then paralyzed with an intraperitoneal injection of 0.2 μg/gm pancuronium bromide (Hospira Inc., Lake Forest, IL, United States). After a standard volume history and two total lung capacity maneuvers, small volume amplitude oscillations at a frequency of 0.9 Hz were applied at a constant volume to the airway opening for 16 s and respiratory system resistance (Rrs) was calculated with the FlexiVent 5.0 software package. The animals were then challenged with an ultrasonic aerosol of PBS containing 0, 3, 6, 12, and 24 mg/ml of methacholine for 10 s each. For 5 min intervals, measurements of airway resistance were again obtained using the forced oscillation technique every 30 s and Rrs calculated. The peak Rrs with each dose was used to generate a dose response curve.

### Immunofluorescent Cytology

Human bronchial smooth muscle cells were grown to 70% confluence in T75 tissue culture flasks. These cells were trypsinized and cytocentrifuged (200 ×*g* for 10 min), and then fixed with acetone. Cytocentrifuge samples were then hydrated and incubated with FC Block (eBioscience; San Diego, CA, United States) (1:100 diluted in permeabilization Buffer), at RT for 20 min. They were then treated with and Avidin/Biotin blocking kit (Vector Labs; Burlingame, CA, United States) and then incubated with 1:200 dilution R3F1 primary or isotype control antibody at 4°C overnight, followed by a biotin-conjugated anti-mouse secondary antibody (Jackson Immunoresearch; Westgrove, PA, United States) for 1 h. Samples were then washed and incubated with Alexa fluor 488 labeled streptavidin (Invitrogen, Carlsbad, CA, United States) for 1 h. The samples were mounted in an aqueous mounting solution containing DAPI. The samples were examined with a Nikon i80 fluorescent microscope fitted with a low light digital camera and image analysis software.

### Chemicals and Solutions

The PSS used in digital calcium measurement contained the following: Na^+^ 140 mM, K^+^ 5.0 mM, Ca^2+^ 2 mM, Cl^-^ 147 mM, HEPES 10 mM, and glucose 10 mM. For the Ca^2+^-free solution, Ca^2+^ was omitted and 0.5 mM EGTA was added to prevent possible Ca^2+^ contamination. The osmolalities for all solutions were ∼284 mOsm/kg.

### Statistical Analysis

All data were expressed as the means for a series of n experiments ± SE. and performed a normality test. Differences between means were considered to be statistically significant at *P* < 0.05 using Student’s *t*-test or one-way ANOVA followed by Newman–Keuls *post hoc* test, as appropriate. In the case of the RNA time-course, a repeated measures analysis of variance with a Bonferroni *post hoc* test was performed.

## Results

### Expression of NCX1 Proteins in Murine Airways

**FIGURE 1:**
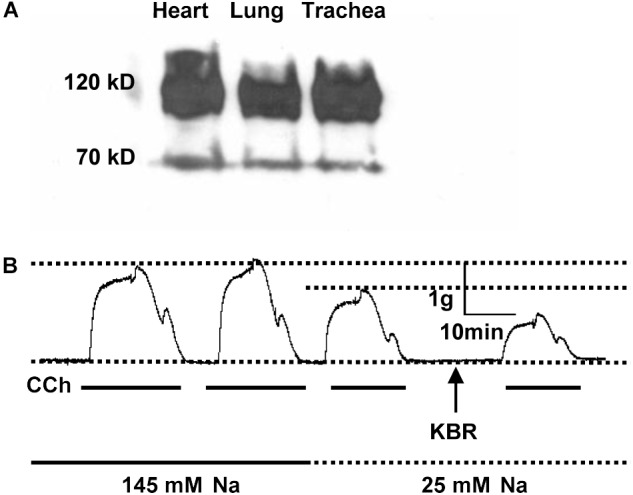
The expression and function of NCX1 proteins in murine airway. **(A)** Proteins were extracted from mouse heart, lung, or trachea tissues, separated by electrophoresis on 4–15% SDS-PAGE, and then transferred to nitrocellulose membranes. The protein-bound nitrocellulose sheets were incubated with R3F1 monoclonal antibody to NCX1 proteins. R3F1 recognized two proteins in murine lung, trachea and heart (as a control) with molecular masses of 120 and 70 kDa. **(B)** Mouse tracheal rings from 3 different mice were placed in an organ bath containing gassed PSS, and then carbachol (CCh, 100 μM)-induced isometric tension was recorded in the presence of 145 mM Na^+^, 25 mM Na^+^, or 25 mM Na^+^ plus KB-R7943 (10 μM). Representative records showing functional role of NCX1 proteins in mediating contraction of murine airway (*n* = 3 different mice for all experiments).

Previous Northern blot analysis on rat tissues demonstrated that NCX1, NCKX3, and NCKX4 mRNA are all expressed in airway smooth muscle. However, NCX2-3 and NCKX1-2 are expressed at an insignificant level in smooth muscle (Visser and Lytton, 2007). These molecular biological data provide evidence for the mRNA expression of NCX isoforms in airway smooth muscle. Since little is known about protein expression of NCXs in the airway, we performed Western blot analyses to detect their protein expression. In the present study, we focused on NCX1 proteins in the murine airway since it is a major isoform of NCXs expressed in mammalian smooth muscle (Liu et al., 2010). R3F1, an anti-NCX1 monoclonal antibody, recognized two proteins in murine lung, trachea and heart (as a control) with molecular masses of 120 and 70 kDa (Figure 1A), corresponding to previous reports of the native NCX1 proteins (Li et al., 2000; Van Eylen et al., 2001). Therefore, NCX1 proteins are abundantly expressed in the murine airway.

### Functional Role of NCX1 Proteins in Mediating Contraction of Murine Airway

Since NCX1 proteins are expressed in the airway, we next assessed NCX activity in the trachea. For this purpose, contractility of the murine tracheal rings was recorded with a multichannel recorder. Carbachol (CCh, 100 μM), a muscarinic receptor agonist, induced two comparable phases of tracheal contraction in normal PSS (Figure 1B, first and second tracings). However, when external Na^+^ was reduced from 145 to 25 mM to inhibit the Ca^2+^ entry mode of NCX, the CCh-induced contraction was attenuated (Figure 1B, third tracing). KB-R7943 (10 μM), a selective inhibitor for the Ca^2+^ entry mode of NCX, further inhibited the CCh-induced tracheal contraction (Figure 1B, fourth tracing). Thus, our data provided evidence for functional expression of NCX1 proteins in the murine airway (Dai et al., 2006; Hirota et al., 2007), consistent with others’ reports that NCX plays a role in regulating airway smooth muscle tone (Dai et al., 2006, 2007; Algara-Suarez et al., 2007; Hirota and Janssen, 2007; Rahman et al., 2012).

### Functional Identification of the Ca^2+^ Entry Mode of NCX1 in Murine Tracheal Smooth Muscle Cells

**FIGURE 2:**
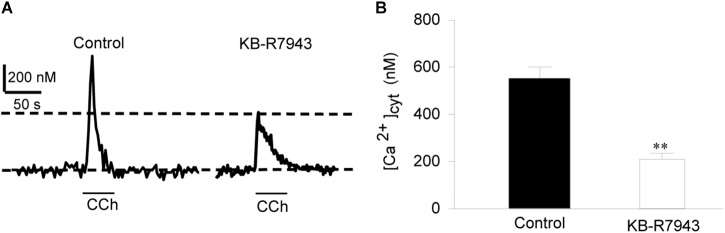
The Ca^2+^ entry mode of NCX contributes to CCh-induced increase in [Ca^2+^]_cyt_ in primary cultures of mouse tracheal single smooth muscle cells. After mouse tracheal smooth muscle cells were loaded with Fura 2-AM, [Ca^2+^]_cyt_ in the cells was measured by digital Ca^2+^ imaging system. **(A)** Representative records showing the time course of carbachol (CCh, 100 μM)-induced [Ca^2+^]_cyt_ changes in mouse tracheal single smooth muscle cells (left panel) and inhibition by 10 μM KB-R7943 (right panel). **(B)** Summarized data showing amplitude of [Ca^2+^]_cyt_ increases in control cells and cells pretreated with KB-R7943. ^∗∗^*P* < 0.01, *n* = 20 cells for each group.

**FIGURE 3:**
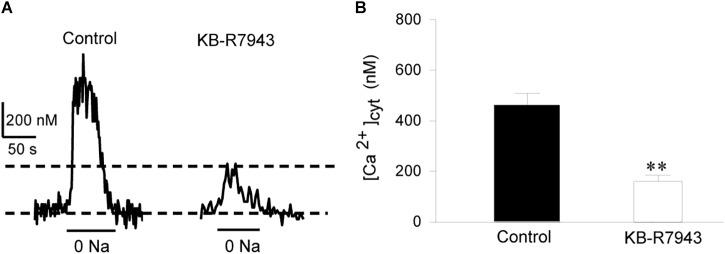
The Ca^2+^ entry mode of NCX contributes to removal of external Na^+^ (0 Na^+^)-induced increase in [Ca^2+^]_cyt_ in individual mouse tracheal smooth muscle cells. After mouse tracheal smooth muscle cells were loaded with Fura 2-AM, [Ca^2+^]_cyt_ was measured by digital Ca^2+^ imaging system. **(A)** Representative records showing the time course of 0 Na^+^-induced [Ca^2+^]_cyt_ changes in individual mouse tracheal smooth muscle cells (left panel) and with inhibition by 10 μM KB-R7943 (right panel). **(B)** Summarized data showing amplitude of [Ca^2+^]_cyt_ increases in control cells and pretreated with KB-R7943. ^∗∗^*P* < 0.01, *n* = 30 cells for each group.

To provide direct evidence for the function of NCX1 in airway smooth muscle cells, primary cultures of mouse tracheal smooth muscle cells were loaded with Fura 2-AM and then [Ca^2+^]_cyt_ in single cells was determined with a digital Ca^2+^ imaging system. CCh (100 μM) induced a transient increase in [Ca^2+^]_cyt_ in mouse tracheal smooth muscle cells (left tracing in Figure 2A and left bar in Figure 2B). To determine the contribution of the Ca^2+^ entry mode of NCX to this response, the cells were pretreated with KB-R7943 (10 μM) and then perfused with same concentration of CCh. KB-R7943 markedly attenuated CCh-induced increase in [Ca^2+^]_cyt_ (right tracing in Figure 2A and right bar in Figure 2B). Removal of extracellular Na^+^ (replaced by equimolar *N*-methyl-D-glucamine) reverses the transmembrane Na^+^ gradient to favor Na^+^ extrusion and Ca^2+^ entry via NCX (Blaustein, 1982; Wen et al., 2016), which induced a rapid increase in [Ca^2+^]_cyt_ (left tracing in Figure 3A and left bar in Figure 3B). These data indicate that the Ca^2+^ entry mode of NCX is active in primary cultures of mouse tracheal smooth muscle cells. KB-R7943 (10 μM) also markedly attenuated the increase in [Ca^2+^]_cyt_ via NCX (right tracing in Figure 3A and right bar in Figure 3B). These data obtained from direct measurement of [Ca^2+^]_cyt_ in primary cultures of mouse tracheal smooth muscle cells have provided further support to our notion that NCX plays a critical role in controlling [Ca^2+^]_cyt_ homeostasis in the airway smooth muscle.

### TNF-α Increases Expression of NCX1 in HBSMCs

**FIGURE 4:**
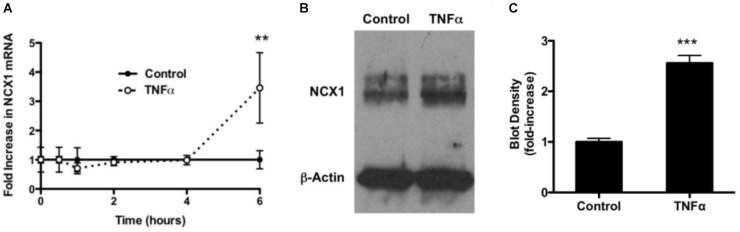
Expression of NCX1 in HBSMCs and enhancement of expression by TNF-α. **(A)** HBSMCs were conditioned without or with 10 ng/ml TNF-α for up to 6 h and then RNA was extracted and analyzed by real time RT-PCR as detailed in Methods. Data were normalized to GAPDH. Cells not conditioned with TNF-α showed no change in mRNA encoding for NCX1. HBSMCs conditioned with TNF-α for showed a 3.5-fold increase in mRNA encoding for NCX1 at 6 h. ^∗∗^*P* < 0.001 vs. control, *n* = 6. **(B)** HBSMCs conditioned without or with 10 ng/ml of TNF-α for 24 h and then proteins were subjected to Western blotting, probed with the NCX1 monoclonal antibody. A representative blot shows that TNF-α-conditioned samples contained significantly more immunoreactive NCX1, but β-Actin did not change. **(C)** Densitometry of summary demonstrating a 2.5-fold increase in TNF-α. ^∗∗∗^*P* < 0.0001 vs. control, *n* = 5.

To determine if TNF-α had an effect on mRNA encoding for NCX1, HBSMCs were cultured in the absence and presence of 10 ng/ml of TNF-α for varying periods of time. Extracted RNA subjected to real-time quantitative RT-PCR revealed that RNA encoding for NCX1 did not change in control samples over 6 h, but, at 6 h, NCX1 mRNA in TNF-α-treated cells had significantly increased 3.5-fold (Figure 4A). GAPDH was used as a normalizing control gene and revealed no change.

To further examine the expression of NCX1 in HBSMCs, cells were again conditioned without or with 10 ng/ml TNF-α for 24 h and proteins then subjected to Western blotting. NCX1 protein was significantly increased in TNF-α cells (Figure 4B). Western blot loading was controlled for by probing with β-Actin. Densitometry of these blots revealed a 2.5-fold increase in NCX1 in TNF-α-conditioned cells relative to controls (Figure 4C). There was no change in actin under the same conditions and probed on the same plot.

**FIGURE 5:**
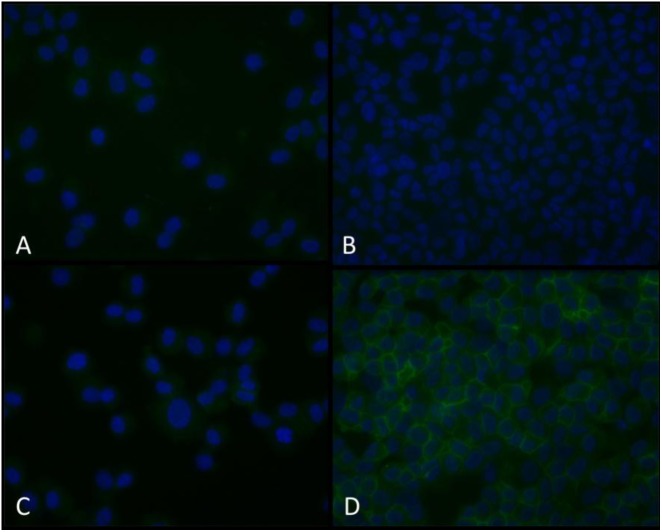
Immunofluorescent NCX1 proteins are increased in TNF-α-conditioned HBSMCs. HBSMCs were grown to near confluence, conditioned without or with 10 ng/ml TNF-α for 24 h and then trypsinized, pelleted on glass slides using a cytocentrifuge, fixed with methanol and probed with a monoclonal antibody to NCX1 as detailed in methods and detected with an Alexa fluor 488 streptavidin-biotin detection system and counter stained with DAPI. **(A)** Control cells with isotype control antibody. **(B)** Control cells with NCX1 antibody. **(C)** TNF-α-conditioned cells with isotype control antibody. **(D)** TNF-α-conditioned cells with NCX1 antibody. Are the cells presented in each panel from similar donors (Patients undergoing lobectomy or lung transplantation for lung cancer who had no evidence of asthma). Extracellular [Ca^2+^] from 2 to 0.2 mM.

To further establish that NCX1 is expressed in HBSMC, cells from similar donors conditioned without and with TNF-α were probed with NCX1 antibody, followed by a biotin-labeled secondary and then Alexa fluor 488-labeled streptavidin. Cells probed with an isotype control antibody revealed no significant fluorescence (Figures 5A,C). Non-conditioned cells probed with NCX1 revealed minimal fluorescence (Figure 5B). However, cells conditioned with 10 ng/ml of TNF-α for 24 h and then probed with NCX1 antibody revealed significant fluorescence in a plasma membrane pattern (Figure 5D).

### Function of NCX1 Proteins and Enhancement by TNF-α in HBSMCs

**FIGURE 6:**
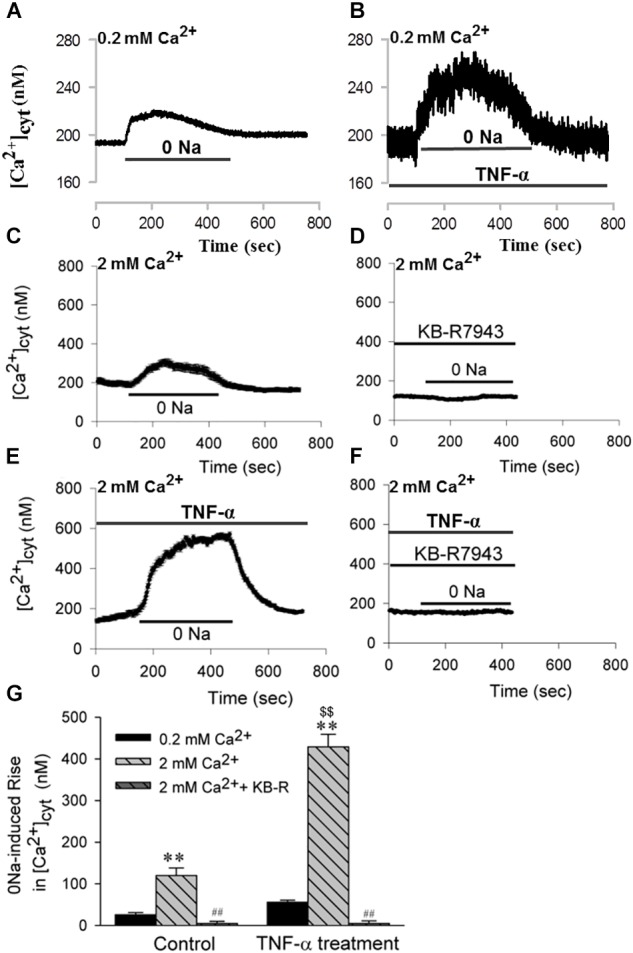
The Ca^2+^ entry mode of NCX1 proteins in HBSMCs and enhancement of function by TNF-α. After HBSMCs were loaded with Fura 2-AM, [Ca^2+^]_cyt_ in the cells was measured by digital Ca^2+^ imaging system. **(A,C)** Removal of Na^+^ induced [Ca^2+^]_cyt_ elevation in normal HBSMC s with extracellular [Ca^2+^] from 0.2 mM **(A)** to 2 mM **(C)**. **(B,E)** 0 Na^+^-induced [Ca^2+^]_cyt_ elevation was enhanced in HBSMCs pretreated with TNF-α (20 ng/ml) for 24 h with extracellular [Ca^2+^] from 0.2 mM **(B)** to 2 mM **(E)**. **(D)** The elevation in panel **C** was prevented by 30 μM KB-R7943. **(F)** The elevation in panel **E** was again prevented by KB-R7943. **(G)** Summarized data showing 0 Na^+^-induced [Ca^2+^]_cyt_ elevation in HBSMCs with different treatments (0.2 mM Ca^2+^, 2 mM Ca^2+^, 2 mM Ca^2+^+KB-R, TNF-α+0.2 mM Ca^2+^, TNF-α+2 mM Ca^2+^, TNF-α+2 mM Ca^2+^+KB-R). ^∗∗^*P* < 0.01 vs. 0.2 mM Ca^2+^; ^##^*P* < 0.001 vs. 2 mM Ca^2+^ without KB-R7943; ^$$^*P* < 0.001 vs. control in 2 mM Ca^2+^, *n* = 30–40 cells for each group.

The next sets of experiments were designed to determine whether HBSMCs express functional NCX1. After HBSMCs were loaded with Fura 2-AM, removal of extracellular Na^+^ (0 Na^+^) induced an increase in [Ca^2+^]_cyt_ (Figures 6A,C). As shown in Figure 6G, decreasing extracellular [Ca^2+^] from 2 to 0.2 mM significantly inhibited (by 10-fold) the 0 Na^+^-induced increase in [Ca^2+^]_cyt_, indicating that the 0 Na^+^-induced rise in [Ca^2+^]_cyt_ is due to inward transportation of Ca^2+^ which is caused by the Ca^2+^ entry model of NCX. Indeed, extracellular application of KB-R7943 (30 μM), a selective inhibitor of the Ca^2+^ entry model of NCX, abolished the increase in [Ca^2+^]_cyt_ via NCX (Figures 6D,G). These data indicate that the Ca^2+^ entry mode of NCX is active in both murine airway smooth muscle cells and HBSMCs.

Furthermore, chronic (or prolonged) treatment of HBSMCs with TNF-α (20 ng/ml, for 24 h) significantly enhanced the NCX activity. As shown in Figure 6B and E the NCX-mediated inward transportation of Ca^2+^, or the 0 Na^+^-induced increase in [Ca^2+^]_cyt_, was much greater in HBSMCs treated with TNF-α, whereas KB-R7943 abolished the NCX-mediated increase in [Ca^2+^]_cyt_ in TNF-α-treated cells (Figures 6F,G). Thus, the proinflammatory cytokine, TNF-α, enhances activity of the Ca^2+^ entry mode of NCX1 proteins and thus may alter [Ca^2+^]_cyt_ homeostasis in the human airway smooth muscle cells.

**FIGURE 7:**
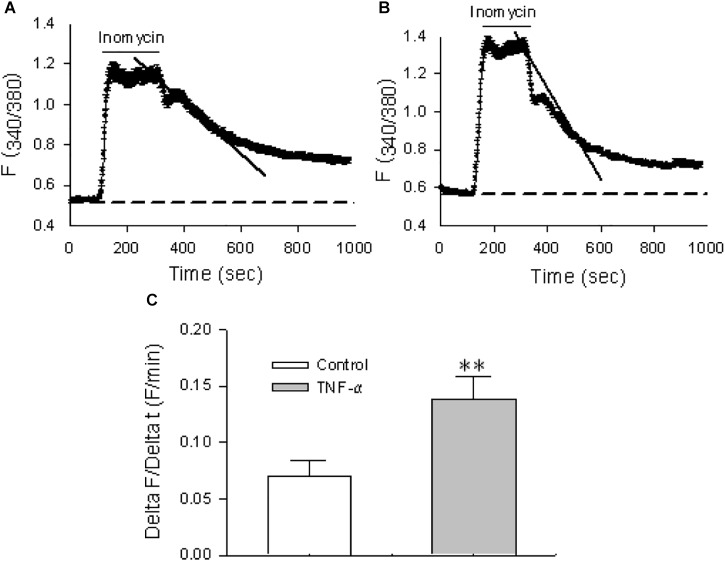
The Ca^2+^ exit mode of NCX1 proteins in HBSMCs and enhancement of function by TNF-α. After HBSMCs were loaded with Fura 2-AM, [Ca^2+^]_cyt_ in the cells was measured by digital Ca^2+^ imaging system. **(A)** In the presence of 30 μM CPA and 100 μM La^3+^, the rate of Ca^2+^ decay was determined after 10 μM ionomycin increased [Ca^2+^]_cyt_ in HBSMCs. **(B)** Same as the protocol in A except HBSMCs were pretreated with TNF-α (20 ng/ml) for 24 h. **(C)** Summarized data showing the rate of Ca^2+^ decay in control HBSMCs or cells pretreated with TNF-α. ^∗∗^*P* < 0.01 vs. control, *n* = 50 cells for each group.

The activity of the Ca^2+^ exit mode of NCX, i.e., the outward transportation of Ca^2+^, was also tested in HBSMCs. To isolate this mode of NCX, cyclopiazonic acid (CPA, 30 μM), a selective inhibitor of the sarco(endo)plasmic reticulum Ca^2+^ pump (SERCA), was used to prevent sequestration of Ca^2+^ into the sarcoplasmic reticulum Ca^2+^ stores and the plasma membrane Ca^2+^ pump (PMCA), or Ca^2+^/Mg^2+^ ATPase, was selectively inhibited by lanthanum (La^3+^, 100 μM) (Shimizu et al., 1997). Thus, when [Ca^2+^]_cyt_ in HBSMCs was raised by ionomycin (10 μM), a Ca^2+^ ionophore, the extrusion of elevated [Ca^2+^]_cyt_ via NCX on the plasma membrane would be the major Ca^2+^ extrusion mechanism under these circumstances (Shimizu et al., 1997). In the presence of CPA and La^3+^ (or when both SERCA and PMCA were inhibited), the rate of Ca^2+^ decay represents activity of the Ca^2+^ exit mode of NCX. As shown in Figure 7A, the Ca^2+^ decay was incomplete because only one of the Ca^2+^ extrusion mechanisms (via the Ca^2+^ exit mode of NCX) remained under these conditions. To test if the proinflammatory cytokine also enhances activity of the Ca^2+^ exit mode of NCX, HBSMCs were pretreated with TNF-α (20 ng/ml) for 24 h. TNF-α significantly increased the rate of Ca^2+^ decay in the presence of CPA and La^3+^ (Figures 7B,C). These data indicate that the proinflammatory cytokine, TNF-α enhances the activity of NCX1 proteins in both the Ca^2+^ exit and entry mode to alter [Ca^2+^]_cyt_ homeostasis in airway smooth muscle cells, suggesting the involvement of the NCX1 proteins in inflammatory diseases of the human airway (White et al., 2006).

### Role of NCX1 Proteins in a Mouse Model of Allergic Airway Inflammation

**FIGURE 8:**
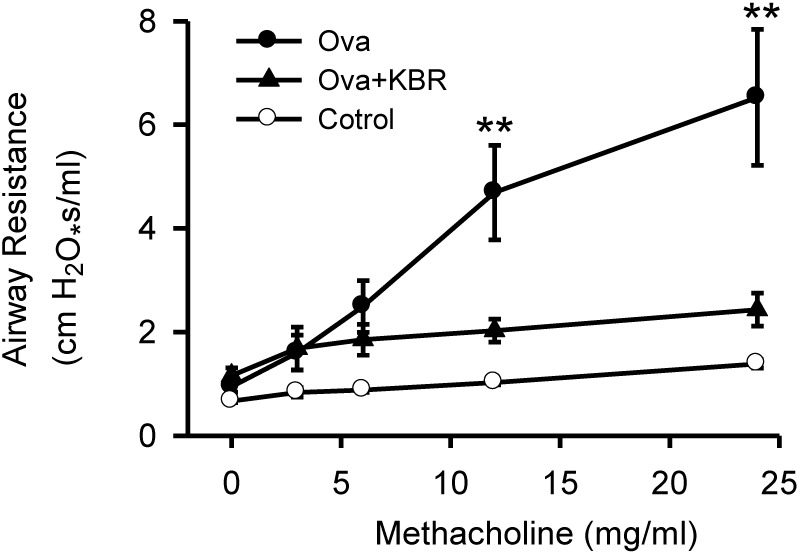
Inhibition of NCX attenuates airway hyperresponsiveness in a murine model of allergic airway inflammation. C57BL/6 mice were immunized and challenged with PBS (control) or ovalbumin in a 21-day model. Thirty min before a methacholine dose-response curve mice received KB-R7943 (10 mg/kg, intraperitoneally) or vehicle. ^∗∗^*P* < 0.001 vs. control, *n* = 12. No statistical differences at all doses between control and KB-R7943-treated ovalbumin mice, *n* = 4.

To further study the role of NCX1 proteins in the airway, we used a well-described ovalbumin mouse model of allergic inflammation and airway hyperresponsiveness. To examine the functional relevance of upregulated expression and activity of NCX1 proteins enhanced in this model, we performed methacholine dose-response curves in control mice, mice immunized and challenged with ovalbumin and in the same mice pretreated with KB-R7943 (10 mg/kg, given intraperitoneally 30 min before study). As illustrated in Figure 8, airway hyperresponsiveness to methacholine was observed in the ovalbumin immunized and challenged mice compared to the control mice without ovalbumin treatment. Moreover, KB-R7943 substantially attenuated the airway hyperresponsiveness to methacholine in ovalbumin immunized and challenged mice (Figure 8), suggesting that NCX1 proteins play a role in airway hyperresponsiveness associated with allergic inflammation in this model.

## Discussion

The present study demonstrates that NCX1 proteins are functionally expressed in the airway and participate in [Ca^2+^]_cyt_ homeostasis in airway smooth muscle cells. Moreover, we show that there is a functional role for NCX1 in both mouse and human airway smooth muscle. Finally we show that the expression and activity of NCX1 proteins are enhanced by proinflammatory cytokine and that the airway hyperresponsiveness to methacholine can be substantially attenuated by KB-R7943, a selective inhibitor of the Ca^2+^ entry mode on NCX1. Our findings are in good agreement with previous reports on the functional expression of NCXs in the airway of animals (Dai et al., 2006, 2007; Algara-Suarez et al., 2007; Hirota and Janssen, 2007; Rahman et al., 2012). We speculate that NCX1 proteins may be involved in the airway hyperresponsiveness observed in asthmatics.

The increase in [Ca^2+^]_cyt_ required to initiate airway smooth muscle contraction may come from both extracellular sites and intracellular stores (Karaki et al., 1997; Santulli and Marks, 2015). Bronchodilation is then achieved by uptake of [Ca^2+^]_cyt_ into the sarco(or endo)plasmic reticulum (S/ER) stores and/or extruding Ca^2+^ across the plasma membrane. [Ca^2+^]_cyt_ homeostasis, particularly in the loading state of the S/ER stores, is controlled by the activity of plasma membrane ion channels and exchangers (Karaki and Weiss, 1984). However, voltage-dependent Ca^2+^ channels do not play a major role in regulating [Ca^2+^]_cyt_ homeostasis and contraction of airway smooth muscle as they do in vascular smooth muscle (Barnes, 1998; Cortijo et al., 2003; Algara-Suarez et al., 2007) and thus currently available classic Ca^2+^ channel blockers have not provided a breakthrough class of therapeutic agents for the treatment of asthma (Middleton, 1985). A growing line of evidence indicates that, in airway smooth muscle, the contractile response is more dependent on Ca^2+^ release from intracellular stores and Ca^2+^ influx through voltage-independent pathways, such as transient receptor potential (TRP) channels and NCXs (Gosling et al., 2005; Nilius et al., 2007; Earley, 2013). While TRP channels have been studied in the airway smooth muscle extensively (Gosling et al., 2005; White et al., 2006; Nilius et al., 2007; Ito et al., 2008; Liu et al., 2010; Earley, 2013), NCXs in the airway have been surprisingly neglected by the asthma field. As discussed below, since a functional coupling of TRPC3 and NCX1 proteins was previously demonstrated in vascular smooth muscle cells (Doleschal et al., 2015; Harper and Sage, 2016), a recent report on the enhancement of TRPC3 by TNF-α in the airway smooth muscle cells prompts us to investigate the role of NCX1 proteins in normal airway smooth muscle and allergic airway inflammation (White et al., 2006).

Despite the fact that NCXs have garnered considerable attention over more than three decades, particularly in the heart, little is currently known about them in the airway. Among the NCX and NCKX proteins that are currently described in mammalian cells (Blaustein and Lederer, 1999; Visser and Lytton, 2007; Khananshvili, 2013), NCX1 was first cloned from cardiac muscle, but has also been shown to be abundantly expressed in many other tissues, including epithelium and smooth muscle (Blaustein and Lederer, 1999). Although there is molecular evidence for NCX1 gene present in human airway smooth muscle (Liu et al., 2010), studies examining regulation by NCX of [Ca^2+^]_cyt_ and the downstream effects on airway smooth muscle contractility have been contradictory. A few functional studies have provided indirect evidence that NCX may be involved (Dai et al., 2006, 2007; Algara-Suarez et al., 2007; Hirota and Janssen, 2007; Rahman et al., 2012); but other studies do not support this conclusion (Knox and Ajao, 1994; Janssen et al., 1997). So far, there is not enough sufficient evidence regarding the protein expression of NCXs in the human airway smooth muscle. Therefore, the current study has provided the further evidence for the expression of NCX1 proteins in human airway smooth muscle cells. But more important is we have revealed that pharmacological inhibition of NCX1 attenuates murine airway hyperresponsiveness observed in an allergic model.

Our functional data clearly reveal an important role of NCX in controlling [Ca^2+^]_cyt_ in airway smooth muscle cells and airway contraction. First, removal of extracellular Na^+^ (0 Na^+^) that reverses the transmembrane Na^+^ gradient for Na^+^ extrusion and Ca^2+^ entry via NCX (Blaustein, 1982; Wen et al., 2016), induces a rapid increase in [Ca^2+^]_cyt_ in primary cultures of mouse and human airway smooth muscle cells. Second, 0 Na^+^-induced [Ca^2+^]_cyt_ signaling depends on extracellular Ca^2+^. Third, 0 Na^+^-induced [Ca^2+^]_cyt_ signaling is sensitive to KB-R7943, a selective inhibitor for the Ca^2+^ entry mode of NCX. Fourth, selective inhibition of the Ca^2+^ entry mode of NCX attenuates an increase in [Ca^2+^]_cyt_ in airway smooth muscle cells and airway contraction induced by the activation of muscarinic receptors. Therefore, since most of the previous studies used tracheal contractility as an indirect measurement of the NCX function, our study by directly measuring the activity of NCXs in airway smooth muscle cells confirms the previous reports on the functional expression of NCXs in normal airway smooth muscle (Dai et al., 2006, 2007; Algara-Suarez et al., 2007; Hirota and Janssen, 2007; Rahman et al., 2012).

Although little is currently known about its possible role in asthma (Sathish et al., 2011), NCX1 has been found to regulate vascular smooth muscle cell proliferation (Zhang et al., 2005), and play an important role in the development of systemic and pulmonary arterial hypertension (Zhang et al., 2007). Thus, it is logical for us to hypothesize their involvement in the pathological process of asthma. To test our hypothesis, we performed several biochemical and functional studies. First, we treated primary cultures of HBSMCs with the proinflammatory cytokine, TNF-α and found that it enhances the expression and activity NCX1 in the cells. Second, we established a well-recognized model of allergic airway inflammation in mice immunized and challenged with ovalbumin and we found that KB-R7943 substantially attenuated hyperresponsiveness to methacholine. All of these results suggest that NCX1 proteins play an important role in the development of airway responsiveness in asthma and that they may represent novel targets for the treatment of asthma.

Na^+^/Ca^2+^ exchangers can operate either in the Ca^2+^ exit mode or in the Ca^2+^ entry mode, depending on the Na^+^ and Ca^2+^ gradients and the potential across the plasma membrane. Although the Ca^2+^ exit mode of NCX plays a major role in expelling elevated [Ca^2+^]_cyt_ from cells, increasing evidence has suggested that the Ca^2+^ entry mode of NCX also contributes to [Ca^2+^]_cyt_ homeostasis (Blaustein and Lederer, 1999; Lee et al., 2001; Zhang et al., 2005). Blaustein (Arnon et al., 2000; Blaustein and Golovina, 2001) and van Breemen (Nazer and Van Breemen, 1998; Lee et al., 2001, 2002; Szado et al., 2003) have recently proposed a working model for Ca^2+^ entry via the entry mode of NCX. Briefly, they propose that stimulation of G protein-coupled receptors either depletes Ca^2+^ stored in the S/ER via PLC-IP_3_ pathway and then opens the SOC, or activates PKC and then opens the receptor-operated channels (ROC). Under physiological conditions, opening of SOC/ROC results mainly in Na^+^ influx into the restricted plasma membrane-S/ER junctional space, causing membrane depolarization. Both the increase in Na^+^ and depolarization drive NCX into the Ca^2+^ entry mode of operation, further bringing Ca^2+^ into the cell. In airway smooth muscle cells, an increase in [Ca^2+^]_cyt_ cannot only trigger Ca^2+^ release from the intracellular Ca^2+^ stores to induce airway smooth muscle contraction, but also may refill the empted intracellular stores with Ca^2+^. A growing line of evidence indicates the physical and functional couplings of TRPC3 and NCX1 proteins (Doleschal et al., 2015; Harper and Sage, 2016), Since TNF-α can enhance both TRPC3-encoded SOC/ROC (Zhang et al., 2005) and NCX1 proteins in the present study, we speculate that they may be activated in concert to participate in the pathological process of asthma, similar to the mechanisms of the involvement of NCX1 proteins in the development of systemic and pulmonary arterial hypertension (Zhang et al., 2007).

In summary, we have confirmed the expression and function of NCX1 in normal human and murine airway smooth muscle cells. We have demonstrated that TNF-α treatment of human airway smooth muscle cells enhances the expression and activity of NCX1. We have also revealed that pharmacological inhibition of NCX1 attenuates murine airway hyperresponsiveness observed in an allergic model. We therefore suggest that NCX1 might be a relevant therapeutic target in inflammatory airway diseases, such as asthma.

## Author Contributions

YW and HD designed the experiment and wrote and finalized the manuscript. JW conducted most experiments and data analysis. XM, BX, TZ, and HG conducted part of experiments. All authors read and approved the final manuscript.

## Conflict of Interest Statement

The authors declare that the research was conducted in the absence of any commercial or financial relationships that could be construed as a potential conflict of interest.

## Abbreviations

[Ca^2+^]_cyt_: cytosolic free Ca^2+^ concentrations
CCh: carbachol
HBSMCs: human bronchial smooth muscle cells
NCX1: Na^+^/Ca^2+^ exchanger 1
S/ER: sarcoplasmic or endoplasmic reticulum
SOC: store-operated channels
